# Pre-clinical undergraduate students’ perspectives on the adoption of virtual and augmented reality to their dental learning experience: A one-group pre- and post-test design protocol

**DOI:** 10.12688/f1000research.53059.2

**Published:** 2023-01-05

**Authors:** Kelvin I. Afrashtehfar, Jing-Wen Yang, A. Al-Sammarraie, Hui Chen, Musab H. Saeed

**Affiliations:** 1 Department of Clinical Sciences, College of Dentistry, Ajman University, Ajman City, PO Box 346, United Arab Emirates; 2 Department of Reconstructive Dentistry & Gerodontology, School of Dental Medicine, Faculty of Medicine, Universität Bern, Bern, 3010 Bern, Switzerland; 3 Department of Prosthodontics, Peking University School and Hospital of Stomatology, National Engineering Laboratory for Digital and Material Technology of Stomatology, Research Center of Engineering and Technology for Digital Dentistry of Ministry of Health, Beijing Key Laboratory of Digital Stomatology, Beijing, 100081, China; 4 Division of Restorative Dental Sciences, Faculty of Dentistry, The University of Hong Kong, Prince Philip Dental Hospital, Sai Ying Pun, Hong Kong SAR, China

**Keywords:** Augmented Reality, Curriculum, Dental Students, Health Education, Immersion, Learning, Perception, Virtual Reality.

## Abstract

**Background:** We live in a time where traditional education has rapidly incorporated online modalities due to the recent SARS-CoV-2 (COVID-19) safety measures such as social distancing. Regardless of these challenges, health education constantly strives to implement the best technologies available for an effective student deep learning outcome. Virtual (VR) and augmented reality (AR) in the dental pre-clinical stage may help stimulate students to better understand the foundation material prescribed in the curriculum. Most visual material available for students is still mainly based on 2D graphics. Thus, this study will attempt to evaluate the students' perceptions about implementing VR/AR technologies in the learning setting.

**Methods**: A single-group pretest-posttest design will be implemented where students will be exposed to VR/AR and fill out two questionnaires, one before and one after the exposure.

**Conclusions:** This project is intended to start once the institutional ethical approval is obtained. It is expected that the analysis from the current project will provide recommendations to improve the students' academic curriculum pre-clinical experience. The recommendations will be provided in the form of at least three scientific publications, with one publication for each subject area intended to be evaluated (i.e., head and neck anatomy, dental anatomy, and removable prosthodontics).

## Introduction

Digital technology has been adopted rapidly in student education. Virtual reality (VR) and augmented reality (AR) tools seem to result in an intense immersion ^1^. The spatial perception about 3D objects may reproduce successful representation in a VR environment. However, to date, dental education is primarily aided by 2D image sources. It is believed that a VR teaching environment may deepen the understanding of dental anatomy ^2^. In fact, a teaching environment with spatial representations improves the outcomes compared with 2D graphics, by keeping students motivated and providing them with an improved understanding ^3, 4^.

The study will be conducted on United Arab Emirates (UAE) pre-clinical dental students. It is essential to evaluate the potential for adopting a VR teaching environment for UAE pre-clinical dental students when understanding and learning about each tooth's characteristics, head and neck components, as well as partial and complete removable dental prostheses. It is equally important to assess the impact of VR in these courses, as perceived by the students. Thus, we hypothesized that students who learned using the traditional 2D teaching pre-clinical content (dental anatomy, head and neck, and removable prosthodontics courses) would not perceive a significant advantage in learning while implementing a VR/AR teaching environment.

### Objectives

This study aims to evaluate the dental students’ perceptions of the current undergraduate dental anatomy, head and neck anatomy, and removable prosthodontics training curriculum, as well as their perspectives on the incorporation of virtual learning into the curriculum.

The research questions that the project intends to answer are as follows:

- What are the dental students’ perceptions on the current undergraduate dental anatomy training?
  ○ What are the dental students’ perceptions of the undergraduate dental anatomy training teaching during the 2019 and 2020 summer session (fully online version) versus the 2021 fall session (blended learning)?
- What are the dental students’ perspectives on incorporating virtual learning into the dental anatomy training curriculum?
  ○ What are the dental students’ perspectives of the incorporation of virtual learning into the dental anatomy training curriculum after experiencing the test?
- What are the dental students’ perceptions on the current undergraduate removable prosthodontics training curriculum?
  ○ What are the dental students’ perspectives of the incorporation of virtual learning into the removable prosthodontics training curriculum after experiencing the test?

## Protocol

During the 2022–2023 academic year, we will conduct a study with undergraduate students who have previously taken the evaluated courses.

### Participants

The eligible students (chosen based on the following selection criteria) will be recruited via email and will obtain their consent documents via email. Electronic signatures will be required for indicating consent to participate. The participants will be sent the link to the electronic surveys (pre- and post-test self-administered questionnaires) via email. Copies of the informed consent form and research instruments are available in *Extended data* ^5^.

***Inclusion criteria***. Ajman University second-year undergraduate dental students who took the dental anatomy, head and neck anatomy, and removable prosthodontics courses will be eligible to participate.

***Exclusion criteria***. Students who have not taken the dental anatomy course in the last academic year will not be eligible to participate.

### Data collection

Given the nature of the pretest-posttest design of this study, the prospective data will be collected at two moments ^6^. However, in this case, the same electronic form will be used to collect the data without disconnecting the session. This measure avoids the need to use two independent forms to apply the two questionnaires. Most importantly, the students will not be assigned an individual code to enter in both forms and risking the identification of the students.

The first step consists of an online (host site TBD) questionnaire (see research instrument A in *Extended data* ^5^) to obtain students' perceptions regarding the traditional curriculum of dental anatomy, head and neck, and removable prosthodontics courses, and to determine students’ potential acceptance of virtual learning.

The VR dental learning environment (i.e., use of VR glasses running the free-trial software [ Head & Neck Anatomy version 3.0, 3D Tooth Atlas version 9.0, Complete Dentures version 1.0, and Removable Partial Dentures version 1.0; eHuman Inc., Fremont, Calif., USA] from their own mobile phones) will be available to the pre-clinical students for 30 minutes each, to test the student curriculum's appropriateness.

After exposure to the VR/AR learning environment, the participants will provide feedback (see research instrument B in *Extended data* ^5^). The post-test questionnaire will consist of selecting an answer for each prompt addressing their VR/AR experience and comparing it to their previous teaching methods when the courses were taken. Most answers will be categorical. More detailed description will follow upon completion of the actual study (IREB approved).

### Data analysis

The results will be compiled and analyzed statistically to be presented as figures and tables.

***Statistical analysis***. A statistical software (IBM SPSS Statistics, Version 27.0., IBM Corp.) will be used to perform all the statistical analyses. The variables will be class, gender, age, nationality, and type of high school. The demographic characteristics will be presented in tables.

The frequency of answers for each survey question that uses the "Likert scale" will be assessed with an independent t-test. The development of the self-report survey tool includes an effort in maximizing validity and controlling for response biases (e.g., social desirability and acquiescent) in Likert scales ^7^. The data from each of the examined curricula will be represented as mean and standard deviations. Non-parametric tests, such as the Kruskal-Wallis test and the Mann-Whitney U test, might be used for comparing ordinal variables, domains, and items between courses and academic years. In the case of multiple comparisons between academic years, the Bonferroni correction might be applied. In the case of multiple comparisons between the teaching courses in different academic years, the Bonferroni correction could also be applied.

***Sample size calculation based on convenience sampling***. G*Power software (ver. 3.1.9.7; Heinrich-Heine-Universität Düsseldorf, Düsseldorf, Germany) was used for sample size determination and power analysis ^8^. Hence, it was estimated that 132 or more measurements/surveys are needed to have a confidence level of 95% that the real value is within ±5% of the measured/surveyed value since one batch population size is approximately 200.

### Data safety and storage

The security and confidentiality of the participants’ identities and electronic data files will be protected, and we will keep the data in encrypted files on a password-protected laptop computer. Electronic data will also be kept, for backup purposes, on a password-protected and encrypted external hard drive, and all non-electronic data will be stored in the locked office of a researcher (KIA or MHS).

All printed material will also be stored in a locked cabinet. Since all data will be de-identified, this will be publicly available indefinitely in a secure cloud-based repository such as Mendeley Data.

### Formulation of the recommendations

The researchers (KIA and MHS) will describe and provide recommendations based on the current pre-clinical curriculum for accepting or rejecting the consideration of virtual learning as a learning tool in the pre-clinical setting.

### Dissemination of the results

It is expected that the analysis from the current project will provide recommendations to improve the students' academic curriculum pre-clinical experience in the form of three publications in peer-reviewed Scopus-indexed journals ^9, 10^. This will be one publication for each subject intended to be evaluated (i.e., head and neck anatomy, dental anatomy, and removable prosthodontics). Additionally, we aim to produce at least one publication at a conference proceeding. This process may take less than 12 months from the moment of the ethical approval ( Table 1).

**Table 1.** Timeline chart of the research project tasks.

No.	Tasks						Months						
		1	2	3	4	5	6	7	8	9	10	11	12
1	*Protocol preparation*												
2	Data collection												
3	Data analysis												
4	Initial draft												
5	Final draft												
6	Submissions for publication												

The implementation activities will include giving support to other dental institutions interested in including VR/AR resources in their curricula, such as The University of Hong Kong and The Peking University.

### Ethics statement

The project involves humans considered a vulnerable population (i.e., undergraduate students) who are being exposed to a sensitive question, that is, to report their nationality. Nevertheless, students will have the option not to answer any of the questions or leave the study at any point. In fact, there are no benefits or penalization to the student's grades whether they decide to participate or not. Moreover, this is considered to be a minimal risk study. To avoid influencing the student's participation in response bias, an independent third party will collect the data, and any identifiable data will not be accessed until the course grades have been finalized. The informed consent will clearly state that participating in such a study has no direct educational benefit to the current students who already completed the course. Lastly, the nature of this study proposal requires Institution Research Ethics Review Board (IREB) approval before being conducted. The IREB application will be submitted in the summer of 2021.

### Study status

This research project is intended to start in January 2023 asthe IREB approval has successfully been obtained.

## Discussion

The rapid incorporation of online (distance) learning modalities to traditional (face-to-face) education has become more visible in the last years. Due to SARS-CoV-2 (COVID-19) pandemic, the recent mandatory safety measures, such as social distancing, have abruptly extended the online learning modalities to most academic institutions. Apart from blended learning, health education has constantly strived to implement the most sophisticated technologies available for an effective student deep learning outcome ^1– 4, 11, 12^. The current project adds an innovative perspective to the advancement of health education since 1) most visual material available for students is still mainly based on 2D graphics, and 2) implementing VR/AR technologies in the learning setting has never been more feasible.

By introducing VR/AR to the dental pre-clinical traditional, blended, and fully online learning contexts, we hope to stimulate students to better understand the foundation material prescribed in the dental undergraduate curriculum, as well as have an influence in the curriculum's continuing improvement ^13^.

### Study strengths and limitations

One of the study's strengths is that, most likely, we will meet the sample requirement according to the power calculation described in the data analysis section. Thus, it is expected to include a representative Middle Eastern geographically rich sample (i.e., primarily Israel, Iraq and Syria) and transfer our findings to other Middle Eastern settings.

Regarding the proposal's limitations, the questionnaires to be used to assess students’ perspectives will not be validated. However, there is an important overlap with similar previous studies ^2– 4^.

Future studies could also test students’ overall academic performance ^14^ to their specific course performance when experiencing a traditional set-up versus VR/AR learning environment. However, the possibility of securing the students anonymity might be compromised.

### Implications for practice and research

This study could inform Asian dental educators of the feasibility of implementing AR/VR technologies to determine the effectiveness in a pre-clinical curriculum before expanding its use.

### Study replication potential

This project has been designed and originally planned to be conducted in the UAE (Dubai and Ajman). However, this project can also be replicated in China (Beijing and Hong Kong) as the coauthors have recognized the value of such an educational project and, in fact, they have submitted their respective IREBs.

## Data Availability

No underlying data are associated with this article. Mendeley Data: Undergraduate students responses to VR/AR in their dental education. http://dx.doi.org/10.17632/2kycm5wwgt.1 ^5^. This project contains the following extended data: - Informed Consent Form - Extended.pdf (consent form). - A pre-test questionnaire-sample.pdf (research instrument A). - A post-test questionnaire-sample.pdf (research instrument B). Data are available under the terms of the Creative Commons Attribution 4.0 International license (CC-BY 4.0).
