# Supplementary material for: Pre-clinical undergraduate students’ perspectives on the adoption of virtual and augmented reality to their dental learning experience: A one-group pre- and post-test design protocol
Source: F1000Res. 2023 Jan 5;10:473. Originally published 2021 Jun 15. [Version 2] doi: 10.12688/f1000research.53059.2 (PMC9837452; doi:10.12688/f1000research.53059.2)
Supplement: Peer review history

# Peer review: Reviewer response for version 2

**DOI:** 10.5256/f1000research.141825.r159254

**Published:** 2023-01-12

**Authors:** Anand Marya

**Affiliations:** 1 Department of Orthodontics, Faculty of Dentistry, University of Puthisastra, Phnom Penh, Cambodia; 2 Center for Transdisciplinary Research, Saveetha Dental College , Saveetha Institute of Medical and Technical Science, Saveetha University, Chennai, Tamil Nadu, India

I am satisfied with all the changes made and the paper can be passed to the next stage of indexing.

Is the study design appropriate for the research question?

Yes

Is the rationale for, and objectives of, the study clearly described?

Yes

Are sufficient details of the methods provided to allow replication by others?

Yes

Are the datasets clearly presented in a useable and accessible format?

Yes

Reviewer Expertise:

Orthodontics, Oral Health, Dental education

I confirm that I have read this submission and believe that I have an appropriate level of expertise to confirm that it is of an acceptable scientific standard.

# Peer review: Reviewer response for version 2

**DOI:** 10.5256/f1000research.141825.r159255

**Published:** 2023-01-06

**Authors:** Kenneth Y. T. Lim

**Affiliations:** 1 Office of Education Research, National Institute of Education, Singapore, Singapore

I thank the authors for taking time to understand the intent behind the suggestions. I have no further comments to make.

Is the study design appropriate for the research question?

Yes

Is the rationale for, and objectives of, the study clearly described?

Yes

Are sufficient details of the methods provided to allow replication by others?

Yes

Are the datasets clearly presented in a useable and accessible format?

Yes

Reviewer Expertise:

the learning sciences, pedagogy / andragogy, the use of VR / AR in learning

I confirm that I have read this submission and believe that I have an appropriate level of expertise to confirm that it is of an acceptable scientific standard.

# Peer review: Reviewer response for version 1

**DOI:** 10.5256/f1000research.56403.r153097

**Published:** 2022-10-19

**Authors:** Kenneth Y. T. Lim

**Affiliations:** 1 Office of Education Research, National Institute of Education, Singapore, Singapore

I am grateful for the opportunity to review your manuscript, which describes the perspectives of pre-clinical students with respect to the use of VR / AR within their curriculum.

 The plan for your study is coherently presented to your readers. You may wish to perhaps consider a more explicit review of the extant literature, so that the research questions may be justified in a more contextualised manner.

 You may also wish to include a brief section on any anticipated risks, and any consequent (tentative) measures you anticipate you might be able to take, should the need arise to mitigate risks.

*Is the rationale for, and objectives of, the study clearly described?*

 The objectives are explicitly stated.

 The rationale might be further improved by situating it more clearly within the literature. the authors are invited to elaborate on their understanding (as derived from literature) of the possible connections between the introduction of a spatial component in the dentistry curriculum through VR / AR and any resulting improvement in learner skillset.

*Is the study design appropriate for the research question?*

 The study design is appropriate for the research questions.

*Are sufficient details of the methods provided to allow replication by others?*

 The description of methods is currently not sufficiently specific. given that the study is yet to be conducted, an argument can reasonably be made that a more detailed description will follow upon completion of the actual study (which is pending IRB approval).

*Are the datasets clearly presented in a useable and accessible format?*

 The question of datasets is not relevant to this manuscript, as the manuscript represents a proposed study and not one which has already been completed.

Is the study design appropriate for the research question?

Yes

Is the rationale for, and objectives of, the study clearly described?

Yes

Are sufficient details of the methods provided to allow replication by others?

Yes

Are the datasets clearly presented in a useable and accessible format?

Yes

Reviewer Expertise:

the learning sciences, pedagogy / andragogy, the use of VR / AR in learning

I confirm that I have read this submission and believe that I have an appropriate level of expertise to confirm that it is of an acceptable scientific standard.

# Response

**Published:** 2022-12-03

**Authors:** Kelvin Afrashtehfar

**Affiliations:** Ajman University, United Arab Emirates

Reviewer #2 is thanked for providing suggestions for improving this manuscript. The positive comments about the completeness of the study protocol are appreciated.

 1.- It is already mentioned that "this is considered a minimal risk study." However, it was added that students are considered a vulnerable population. 

 Thus, the data collected does not reveal their identity, and anonymity is always preserved. Additionally, they are free to participate or withdraw from the study at anytime. There are no benefits or penalization to the student's grades whether they decide to participate or not. To avoid influencing the student's participation in response bias, an independent third party will collect the data, and any identifiable data will not be accessed until the course grades have been finalized.

 The informed consent will clearly state that participating in such a study has no educational benefit to the students.

 2.- The rationale or justification of the appraised study is mentioned as "It is essential to evaluate the potential for adopting a VR teaching environment for UAE pre-clinical dental students when understanding and learning about each tooth's characteristics, head and neck components, as well as partial and complete removable dental prostheses. It is equally important to assess the impact of VR in these courses, as perceived by the students." A hypothesis has also been provided.

 The implications "Implications for practice and research" agree and connect the introduction rationale with the discussion by stating, "This study could inform Asian dental educators of the feasibility of implementing AR/VR technologies to determine the effectiveness in a pre-clinical curriculum before expanding its use." 

 3.- The author is thanked for providing a solution for addressing additional specifics regarding the methods. Thus, the reviewer's suggestion has been added to the manuscript text. "More detailed description will follow upon completion of the actual study (IREB approved)."

 The sentence "This research project is intended to start once the IREB approval is obtained (fall 2021)" was changed to "This research project is intended to start in January 2023 as the IREB approval has been obtained."

 The authors acknowledge reviewer #2 for providing comments to improve the submitted manuscript. We are delighted with the result.

# Peer review: Reviewer response for version 1

**DOI:** 10.5256/f1000research.56403.r137499

**Published:** 2022-06-22

**Authors:** Anand Marya

**Affiliations:** 1 Department of Orthodontics, Faculty of Dentistry, University of Puthisastra, Phnom Penh, Cambodia; 2 Center for Transdisciplinary Research, Saveetha Dental College , Saveetha Institute of Medical and Technical Science, Saveetha University, Chennai, Tamil Nadu, India

First of all I would like to thank the editor for inviting me to review this paper and secondly I would like to complement the authors on their hard work in compiling this manuscript. I do have some suggestions to help improve this manuscript: 

1. Please do mention the mode of sampling which I think in this case is convenience sampling.
2. Is the post-test questionnaire blinded? Bias elimination as it is a single-center study.
3. What software/method has been used for sample size calculation and please expand on the power of the sample.
4. Please add notes on limitations of Likert scale.
5. Please consider the following papers:
  a. Jaber M, Al-Samarrai B, Al-Obaidee A, Varma SR, Karobari MI, Marya A. Does General and Specific Traits of Personality Predict Students’ Academic Performance?. BioMed Research International. 2022 Jan 4;2022.
  This is an insightful study into the academic performance of students based on their traits which are very important factors to consider for any educational perspective study.
  b. Veeraiyan DN, Varghese SS, Rajasekar A, Karobari MI, Thangavelu L, Marya A, Messina P, Scardina GA. Comparison of Interactive Teaching in Online and Offline Platforms among Dental Undergraduates. International Journal of Environmental Research and Public Health. 2022 Mar 8;19(6):3170.
  This an undergraduate level study where an analysis is done on the comparison between interactive teaching using various platforms.

Is the study design appropriate for the research question?

Yes

Is the rationale for, and objectives of, the study clearly described?

Yes

Are sufficient details of the methods provided to allow replication by others?

Yes

Are the datasets clearly presented in a useable and accessible format?

Yes

Reviewer Expertise:

Orthodontics, Oral Health, Dental education

I confirm that I have read this submission and believe that I have an appropriate level of expertise to confirm that it is of an acceptable scientific standard, however I have significant reservations, as outlined above.

## References

[1] : Does General and Specific Traits of Personality Predict Students' Academic Performance?. *Biomed Res Int* .2022;2022: 10.1155/2022/9422299 9422299 10.1155/2022/9422299 35039794PMC8761063

[2] : Comparison of Interactive Teaching in Online and Offline Platforms among Dental Undergraduates. *International Journal of Environmental Research and Public Health* .2022;19(6) : 10.3390/ijerph19063170 10.3390/ijerph19063170 PMC895567235328858

# Response

**Published:** 2022-12-03

**Authors:** Kelvin Afrashtehfar

**Affiliations:** Ajman University, United Arab Emirates

The reviewer is acknowledged for providing suggestions for improving this manuscript. The authors have enjoyed reading and addressing every comment of the reviewer’s report.

 1. 'Convenience sampling' was added.

 2. The post-test questionnaire will be answered after the exposure using the same form used for the pre-test questionnaire. This has been added, too.

 3. G*Power software (ver. 3.1.9.7; Heinrich-Heine-Universität Düsseldorf, Düsseldorf, Germany) will be used for sample size determination and power analysis. This has been included and cited.

 Reference: Kang H. Sample size determination and power analysis using the G*Power software. J Educ Eval Health Prof. 2021;18:17. doi: 10.3352/jeehp.2021.18.17.

 4. To comply with the reviewer's comment, "The development of the self-report survey tool includes an effort in maximizing validity and controlling for response biases (e.g., social desirability and acquiescent) in Likert scales," t was added.

 Reference: Kreitchmann RS, Abad FJ, Ponsoda V, Nieto MD, Morillo D. Controlling for Response Biases in Self-Report Scales: Forced-Choice vs. Psychometric Modeling of Likert Items. Front Psychol. 2019 Oct 15;10:2309. doi: 10.3389/fpsyg.2019.02309.

 5. The suggested references Jaber et al. 2022 and Veeraiyan et al. 2022 were revised as indicated. The listed authors decided by consensus that the following references are pertinent to comply with the reviewer's suggestions.

 References: 

 Veeraiyan DN, Varghese SS, Rajasekar A, Karobari MI, Thangavelu L, Marya A, Messina P, Scardina GA. Comparison of Interactive Teaching in Online and Offline Platforms among Dental Undergraduates. International Journal of Environmental Research and Public Health. 2022 Mar 8;19(6):3170.

 Lin GSS, Tan WW, Afrashtehfar KI. Exploring the Learning Experience of High-Performing Preclinical Undergraduate Dental Students: A Qualitative Study. Education Sciences. 2022; 12(11):801. https://doi.org/10.3390/educsci12110801

 The authors appreciate the reviewer's intellectual effort and personal time to provide recommendations to benefit the appraised manuscript and the educational research community.
